# GaWRDenMap: a quantitative framework to study the local variation in cell–cell interactions in pancreatic disease subtypes

**DOI:** 10.1038/s41598-022-06602-z

**Published:** 2022-03-08

**Authors:** Santhoshi N. Krishnan, Shariq Mohammed, Timothy L. Frankel, Arvind Rao

**Affiliations:** 1 Department of Electrical and Computer Engineering, Rice University, Houston, TX USA; 2 Department of Computational Medicine and Bioinformatics, University of Michigan, Ann Arbor, MI USA; 3 Department of Biostatistics, University of Michigan, Ann Arbor, MI USA; 4 Department of Surgery, University of Michigan, Ann Arbor, MI USA; 5 Department of Radiation Oncology, University of Michigan, Ann Arbor, MI USA; 6 Department of Biomedical Engineering, University of Michigan, Ann Arbor, MI USA; 7 Present Address: Department of Biostatistics, Boston University, Boston, MA USA

**Keywords:** Cancer microenvironment, Bioinformatics, Biomedical engineering, Fluorescence imaging

## Abstract

Spatial pattern modelling concepts are being increasingly used in capturing disease heterogeneity. Quantification of heterogeneity in the tumor microenvironment is extremely important in pancreatic ductal adenocarcinoma (PDAC), which has been shown to co-occur with other pancreatic diseases and neoplasms with certain attributes that make visual discrimination difficult. In this paper, we propose the GaWRDenMap framework, that utilizes the concepts of geographically weighted regression (GWR) and a density function-based classification model, and apply it to a cohort of multiplex immunofluorescence images from patients belonging to six different pancreatic diseases. We used an internal cohort of 228 patients comprised of 34 Chronic Pancreatitis (CP), 71 PDAC, 70 intraductal papillary mucinous neoplasm (IPMN), 16 mucinous cystic neoplasm (MCN), 29 pancreatic intraductal neoplasia (PanIN) and 8 IPMN-associated PDAC patients. We utilized GWR to model the relationship between epithelial cells and immune cells on a spatial grid. The GWR model estimates were used to generate density signatures which were used in subsequent pairwise classification models to distinguish between any two pairs of disease groups. Image-level, as well as subject-level analysis, were performed. When applied to this dataset, our classification model showed significant discrimination ability in multiple pairwise comparisons, in comparison to commonly used abundance-based metrics, like the Morisita-Horn index. The model was able to best discriminate between CP and PDAC at both the subject- and image-levels. It was also able to reasonably discriminate between PDAC and IPMN. These results point to a potential difference in the spatial arrangement of epithelial and immune cells between CP, PDAC and IPMN, that could be of high diagnostic significance. Further validation on a more comprehensive dataset would be warranted.

## Introduction

There are multiple disorders that affect the pancreas with various degrees of severity and intensity. Pancreatic ductal adenocarcinoma (PDAC) is an extremely aggressive form of pancreatic cancer, accounting for about 3% of all cancers in the US and 7% of all cancer deaths^1^. Prediction models estimate that there is a risk of almost 60,430 people being diagnosed with the disease in the year 2021, with the disease estimated to be the second most deadly of all cancers by the year 2030^1,2^. Though survival is higher when detected early, successful treatment planning for the disease remains challenging due to the late detection of the disease mostly at an advanced staged, and frequently accompanied with metastatic spread to other regions of the body^3^. A major challenge associated with the diagnosis of this disease is that it is mostly detected at advanced stages, when treatment options are limited^4^. It has been shown that PDAC can develop from preneoplastic pancreatic intraductal neoplasia (PanIN) or can also be associated with intraductal papillary mucinous neoplasm (IPMN) and mucinous cystic neoplasm^5–7^. An important risk factor associated with PDAC is the prior history of Chronic Pancreatitis (CP), a disease characterized by the inflammation and potential irreversible damage to the constituent cells^8^. Both CP and PDAC are characterized by a dense inflammatory stroma composed of fibroblasts and immune cells, making diagnostic discrimination on biopsy difficult. Studies have also shown that there is a difference in the immune and tumor-stromal interplay in the microenvironment among these diseases^9,10^. There is observed heterogeneity in cellular arrangement and the immune makeup not only between these diseases, but also between patients in the same disease cohort, which has been strongly linked to a difference in disease manifestation and progression^11^. Thus, there is an increasing need to understand the spatial biology and the structure of cellular arrangement of PDAC as well as its precursor conditions and differentiate between them. This would potentially assist in the early identification of patients who may harbor invasive PDAC, for further testing and a potential curative resection^12^.

Quantitative assessment of cell phenotypes in the tumor environment is currently done using simple cell-count measures obtained from pathology images of a biopsy by the pathologist. These images can range from the more commonly accessible Hematoxylin and Eosin (H&E) stained whole slide images, to multiplexed immunohistochemistry (mIHC) and immunofluorescence (mIF)-stained images which capture specific antigen expressions. It has been noted that count-based methods, including both manual (as counted by a human observer i.e. a pathologist) and software-assisted cell counting, do not account for the spatial heterogeneity of the different cell types such as tumor and immune cells. However, spatial information has been shown to be an important prognostic factor in various cancers^13,14^. Spatial immune profiling has become increasingly utilized to understand the cellular and molecular heterogeneity of the disease microenvironment. The emergence of multiplexed imaging technologies such as time-of-flight mass cytometry (CyToF)^15^, mIHC, mIF, and co-detection by indexing (CODEX)^16^ have helped in scaling up identification of multiple antigens present on cells on a given tissue environment. From the point of view of cancer biology, a greater understanding of tumor heterogeneity has been shown to be influential in predicting cancer progression, response to various therapeutic procedures, and prognosis^10,14,17,18^. The Morisita-Horn index has been used in histological image analysis to assess co-localization between tumor and immune cells in breast cancer^19,20^. Similarly, the G-cross, a count-type nearest neighbor distance distribution function, has been utilized in multiple studies to assess pairwise cell interactions, and has been shown to be an independent predictor for patient outcome in non-small cell lung cancer and inflammatory breast cancer, among others^10,14^. Such methods do offer additional information about spatial heterogeneity, but offer limited insight into heterogeneity in specific regions of the disease tissue, and by extension the tumor microenvironment under observation. In a parallel vein, spatial pattern-modeling concepts have been used extensively in environmental sciences and public health to correlate cancer outcome with environmental covariates^21^. Hence, we seek to model cellular interactions across different cell species with a spatial context, to better understand the positional and functional influence of cells of one type in determining the location of cells of another type.

**Figure 1:**
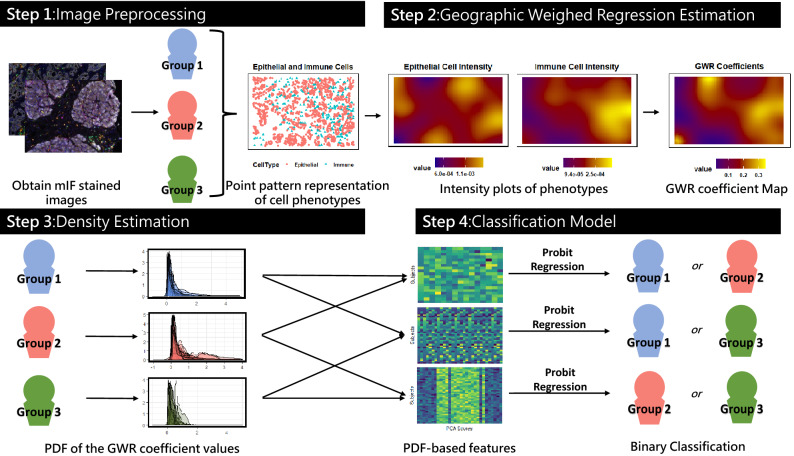
A schema of the proposed GaWRDenMap framework. The framework utilizes the point intensity maps of the cell phenotypes in question to generate a regression map, the values of which are used to construct a density-based feature vector. This vector is then used as an input to a classifier to distinguish between any two groups of diseases.

In this paper, we propose a framework that (i) considers the local variation in distribution of epithelial and immune cells from a pathology image to construct a quantitative (functional) ‘signature’ of pairwise interactions between the cell types, and (ii) use these signatures to discern between subgroups of pancreatic disease. To facilitate this, we represent these cells as a ‘point pattern’, with the ‘point’ location on the spatial grid determined as the center of the cell. We utilize geographically weighted regression (GWR), a spatial regression technique commonly used in ecology and other mapping-related disciplines, to map the spatial variation in epithelial-immune interactions across the slide^22^. This framework is named the GaWRDenMap, where the ’GaWR’ comes from the GWR framework used to generate the regression coefficients, and ’Den’ from the probability density function (PDF) generated from the coefficients obtained. The overall schematic summary of the GaWRDenMap framework is shown in Fig. 1. The framework entails the following steps:

1. Obtain intensity surfaces for the cell phenotype point pattern of interest.
2. Use these intensity surfaces to compute the GWR model coefficients representing relationships between cell phenotypes.
3. Construct a representation of the variation in GWR coefficients across each image via a PDF.
4. Use features derived from the PDF representation as predictors for the classification model.

The paper is organized as follows. The data acquisition, associated pertinent clinical information, and preprocessing steps are explained in the “Data” section. In the “Results” section, the outcomes from our framework and their associated summary statistics are listed and explained, and these results are compared with an existing spatially-aware statistical framework. This is followed with “Discussion” and steps for future work. Finally, in the “Methods” section we conclude with the explanation on how we obtain the intensity surfaces for each patient, and describe how these are used to generate density-based features. We end with a description of the classification model that uses the generated features to categorize the disease subgroups.

## Data

The patients were selected from those presenting to the University of Michigan Pancreatic Cancer Clinic who underwent surgical resection for various pancreatic pathologies. The study was done in accordance with the University of Michigan Institutional Review Board approval. The study population consisted of patients diagnosed wth six different pancreatic diseases, including pancreatic cancer and non-malignant pathologies. This included 34 patients with Chronic Pancreatitis (CP), 29 with Pancreatic intraepithelial neoplasia (PanIN), 16 with Mucinous cystic neoplasm (MCN), 70 with Intraductal Papillary Mucinous Neoplasm (IPMN), 8 with IPMN associated cancers (IPMN-associated PDAC), and 71 with traditional Pancreatic Ductal Adenocarcinoma (PDAC). Normal tissue was not included, as no surgical or oncologic indications currently exist to biopsy non-pathological pancreas which limits our ability to study this tissue type.

Multiplex immunofluorescent staining was performed on a tissue micro-array composed of 0.6mm cores taken from Formalin-fixed Paraffin-embedded (FFPE) tissue blocks in duplicate as has been previously described^23^. Briefly, slides were subjected to serial rounds of antigen retrieval, followed by primary and secondary antibody staining. Tyramide signal amplification was applied with a unique fluorophore for individual antigens. Subsequent antigen retrieval steps cleaved the primary and secondary antibodies leaving the fluorophore in place. Immune cell phenotyping was performed using antibodies to CD3, CD8, CD163, PD-L1, pancytokeratin, and FOXP3. Nuclear staining with DAPI was used to allow discrimination and assign spatial location of individual cells. Associated clinical information was also obtained from patient records. The location coordinates of each cell and its corresponding phenotype label was computed, utilizing user-set thresholds to determine marker expression using Vectra InForm software (Akoya Biosciences) . For this study, epithelial cells were defined as any cell expressing cytokeratin, and an immune cell as any cell expressing CD3 and/or CD8. In PDAC, any cell expressing cytokeratin is considered to be tumorous but is referenced as an epithelial cell throughout the paper for consistency. Both were determined by immune-specific staining. This processing is also done using Inform software (AKOYA Biosciences), for every image in each of the six disease groups. The available demographic and clinical data is shown in Table 1.

**Table 1:** A summary of clinical characteristics of the patient cohort.

Characteristics	CP	PDAC	IPMN	MCN	PanIN	IPMN-associated PDAC
Number of patients	N = 34	N = 71	N = 70	N = 16	N = 29	N = 8
Average number of image slides per patient	1.64	2.01	1.27	1.31	1.41	4.75
Median age at surgery in years (range)	50	64	64	44	63	NA
BMI (Mean SD)	27.16 6.16	28.5 5.61	28.4 7.05	34.05 9.07	23.48 4.56	NA
**Sex**						
Male	13	31	15	0	5	NA
Female	6	29	13	13	7	NA

Missing values were excluded when computing summary statistics in each category.

## Results

We analysed immuno-fluorescence-stained histopathology images belonging to six pancreatic diseases. Within each image we identify two phenotype-specific point pattern windows, one for epithelial cells and the other for immune cells, which were identified through biomarker staining. To ensure that there is a minimum representation of both cell phenotypes in the observation windows for every image, we considered additional inclusion criteria. Firstly, any image not having at least five epithelial and five immune cells was excluded from the analysis. Next, in images where the epithelial point pattern is inconsistent, we threshold the obtained point pattern window using a predetermined threshold value to avoid inconsistency in GWR estimates (see Supplementary Image S2 for more details) . This threshold value is selected to be reasonably small after observing the spread of the intensity values across all images in the cohort. The obtained windows (based on intensity values greater than the threshold) are utilized to segment out the same region from the point intensity of the immune cells. We focus on this common region to compute the GWR model coefficients with epithelial intensity surface as predictor and immune intensity surface as the response.

**Figure 2:**
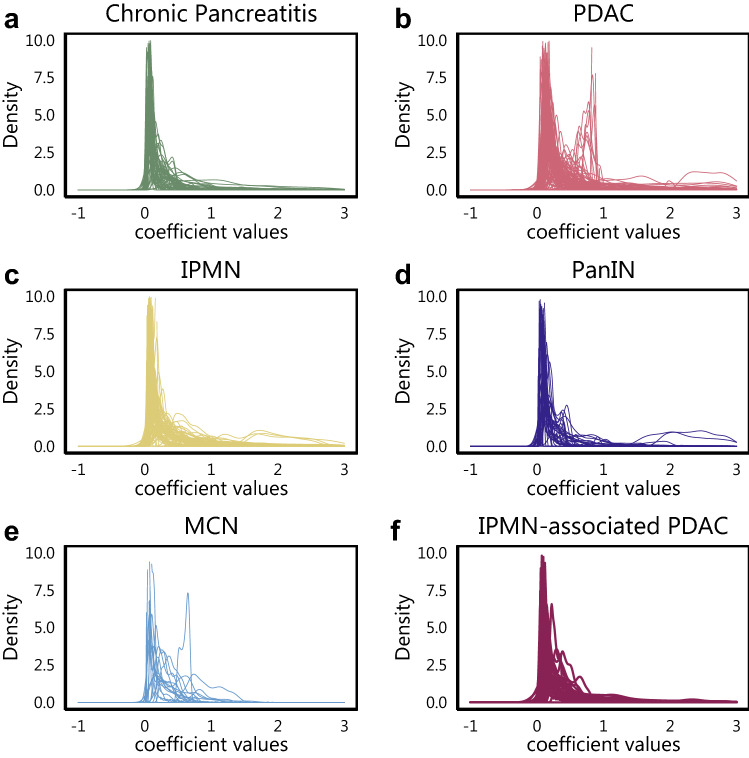
The density estimates for all subjects across all six pancreatic disease subtypes. Similar plots without truncation of the x- and y-axis are shown in Supplementary Fig. S3(online). Here, the x-axis corresponds to the GWR coefficient values obtained at every point on our GWR computation grid.

The GWR model coefficients from the thresholded windows are now used to construct the density representations. We scale the GWR model coefficients between 0 and 1, using their global minimum and maximum to define the range across all the patients. For each image in a disease group, we obtain a PDF constructed using the GWR coefficients, which can be viewed as the ‘signature’ representing the epithelial-immune interaction for the given image or subject. PDFs for all images across all disease groups are shown in Fig. 2. The average of the PDFs for every disease group, represented by the Karcher mean, is also represented along with all the PDFs from each group in Supplementary Fig. S4. We then compute PDF-based features for each group (e.g. PDAC, CP) using a Riemannian-geometric framework (further details in the “Methods” section). Using these PDF-based features as covariates, we build classification models to predict the disease group. We perform Bonferroni correction for multiple pairwise comparisons. We present the corresponding classification area under the receiver operating characteristics curve (AUC) along with associated metrics for all the pairwise comparisons among the six groups. Several subjects had multiple images associated with them, so it was of interest to observe if there would be marked difference in the capabilities of our model between a subject-level and image-level analysis. Subject-level analysis augments the GWR coefficients across all the images for that subject to construct one representative density signature, whereas the image-level analysis treats each image as a standalone sample for the model. We employed this approach for the subject-level analysis, since we do not have access to the relative locations of corresponding image slides on the large biopsy tissue. We computed results for both image- and subject-level analyses using the GaWRDenMap framework, which are shown in Tables 2 and 3, respectively.

**Table 2:** Image-wise classification results between the six pancreatic diseases using GaWRDenMap.

Group 1	Group 2	AUC	AUC CI	Sensitivity	Specificity
Chronic pancreatitis	IPMN	0.614	0.461–0.766	0.75	0.589
Chronic pancreatitis	MCN	0.72	0.503–0.937	0.684	0.732
Chronic pancreatitis	PanIN	0.594	0.417–0.771	0.683	0.589
**Chronic pancreatitis**	**PDAC**	**0.875**	**0.792**–**0.959**	**0.845**	**0.75**
Chronic pancreatitis	IPMN-associated PDAC	0.727	0.577–0.877	0.789	0.589
IPMN	MCN	0.592	0.392–0.792	0.895	0.412
IPMN	PanIN	0.607	0.436–0.779	0.585	0.637
**IPMN**	**PDAC**	**0.753**	**0.652**–**0.854**	**0.744**	**0.688**
IPMN	IPMN-associated PDAC	0.604	0.449–0.759	0.632	0.537
MCN	PanIN	0.666	0.449–0.884	0.634	0.632
**MCN**	**PDAC**	**0.756**	**0.586**–**0.926**	**0.767**	**0.632**
MCN	IPMN-associated PDAC	0.632	0.402–0.861	0.658	0.579
**PanIN**	**PDAC**	**0.808**	**0.682**–**0.934**	**0.845**	**0.756**
**PanIN**	**IPMN-associated PDAC**	**0.767**	**0.611**–**0.923**	**0.763**	**0.659**
PDAC	IPMN-associated PDAC	0.435	0.311–0.56	0.684	0.194

The rows highlighted in bold indicate classification AUCs that are identified as significant, with 0.75 selected as the threshold value for significance. This value was determined after analysing the results as a whole and on consultation with the physician.

**Table 3:** Subject-wise classification results between the six pancreatic diseases using GaWRDenMap.

Group 1	Group 2	AUC	AUC CI	Sensitivity	Specificity
Chronic pancreatitis	IPMN	0.637	0.444–0.83	0.742	0.588
Chronic pancreatitis	MCN	0.674	0.409–0.94	0.571	0.735
Chronic pancreatitis	PanIN	0.621	0.408–0.833	0.69	0.529
**Chronic pancreatitis**	**PDAC**	**0.865**	**0.752**–**0.977**	**0.776**	**0.824**
Chronic pancreatitis	IPMN-associated PDAC	0.684	0.317–1	0.625	0.853
IPMN	MCN	0.618	0.365–0.87	0.571	0.613
IPMN	PanIN	0.536	0.332–0.741	0.586	0.661
**IPMN**	**PDAC**	**0.781**	**0.661**–**0.901**	**0.731**	**0.758**
IPMN	IPMN-associated PDAC	0.554	0.295–0.814	0.75	0.484
MCN	PanIN	0.682	0.395–0.969	0.793	0.714
MCN	PDAC	0.647	0.371–0.924	0.657	0.571
MCN	IPMN-associated PDAC	0.478	0.085–0.87	0.625	0.571
**PanIN**	**PDAC**	**0.79**	**0.635**–**0.944**	**0.791**	**0.724**
PanIN	IPMN-associated PDAC	0.655	0.35–0.96	0.5	0.828
PDAC	IPMN-associated PDAC	0.58	0.311–0.85	0.625	0.642

The rows highlighted in bold indicate classification AUCs that are identified as significant, with 0.75 selected as the threshold value for significance. This value was determined after analysing the results as a whole and on consultation with the physician.

At the image-level (Table 2), the classification model was able to best discriminate between CP versus PDAC with an AUC of 0.875 (0.792–0.959). The model was also able to significantly discriminate IPMN vs PDAC, MCN vs PDAC, PanIN vs PDAC and PanIN vs IPMN-associated PDAC, with corresponding AUCs of 0.753 (0.652–0.854), 0.756 (0.586–0.926), 0.808 (0.682–0.934) and 0.767 (0.611–0.923), respectively. This indicates that we are able to distinguish PDAC from it’s precursor and co-occurring conditions with significant accuracy, when considering each image as a distinct sample. At the subject-level (Table 3), the classification model discriminated best between CP and PDAC with an AUC of 0.865 (0.752–0.977). However, as compared to the image-level classifier, only IPMN vs PDAC and PanIN vs PDAC were found to be significantly discriminable, with corresponding AUCs of 0.781 (0.661–0.901) and 0.79 (0.635–0.944). We observe similarly good discrimination capability in our pairwise classifier in distinguishing PDAC from the precursor conditions, when considering each subject to be a distinct sample. From the results, we observe that there is a reasonable consistency between the image-level and subject-level classification results, and thus doing either subject-level or image-level analysis on a similarly structured data set should have a minimal effect on the overall results. While at the image-level, our approach ignores correlations by considering each image as a separate independent entity, the subject-level analysis accounts for the grouped nature of these images by pooling GWR coefficients from all images within a subject to compute a single density for that subject, in the absence of required spatial data. Additionally, due to GWR being a local spatial regression, the coefficient estimates at any point in one image will likely not be strongly influenced by points from other images of the same subject.

We wanted to explore how our framework does in comparison to previously proposed methodologies that also take into context the spatial arrangement of different cell types in the microenvironment. To this effect, we computed the Morisita-Horn (M-H) index, a spatially informed metric for dissimilarity that has been shown to be prognostic in many diseases including breast cancer^20,24^. The M-H index was computed as a baseline metric for every image in each disease group analysed in this study. Further details on the methodology are given in the supplementary material. We perform similar analysis with the MH-index values as predictors and the results of the pairwise classification models are shown in Table 4. We find that the GaWRDenMap framework outperforms the MH-based classification model for a majority of the disease pairs, with the exception of PDAC and PanIN, with an AUC of 0.577 (0.472–0.682) for the M-H Index based classification model. It is observed that the model performance AUC metrics range between 0.5 to 0.6, with the pairs of CP and IPMN performing barely better than a random classifier.

**Table 4:** Image-wise classification results between the six pancreatic diseases using the Morisita-Horn Index.

Group 1	Group 2	AUC	AUC CI	Sensitivity	Specificity
Chronic pancreatitis	IPMN	0.517	0.419–0.614	0.494	0.607
Chronic pancreatitis	MCN	0.569	0.399–0.739	0.571	0.679
Chronic pancreatitis	PanIN	0.555	0.435–0.674	0.561	0.571
Chronic pancreatitis	PDAC	0.546	0.459–0.632	0.580	0.536
Chronic pancreatitis	IPMN-associated PDAC	0.547	0.423–0.670	0.632	0.571
IPMN	MCN	0.576	0.410–0.741	0.619	0.663
IPMN	PanIN	0.577	0.466–0.688	0.561	0.618
IPMN	PDAC	0.582	0.506–0.658	0.657	0.528
IPMN	IPMN-associated PDAC	0.580	0.469–0.69	0.605	0.618
MCN	PanIN	0.548	0.387–0.710	0.659	0.476
MCN	PDAC	0.589	0.449–0.728	0.657	0.476
MCN	IPMN-associated PDAC	0.598	0.442–0.753	0.579	0.571
PanIN	PDAC	0.610	0.512–0.708	0.636	0.512
PanIN	IPMN-associated PDAC	0.625	0.500–0.748	0.605	0.634
PDAC	IPMN-associated PDAC	0.577	0.472–0.682	0.605	0.608

The rows highlighted in bold indicate classification AUCs that are identified as significant, with 0.75 selected as the threshold value for significance.This value was determined after analysing the results as a whole and on consultation with the physician. **No row was highlighted in this case, as no AUC value was above the 0.75 threshold mark.**

**Figure 3:**
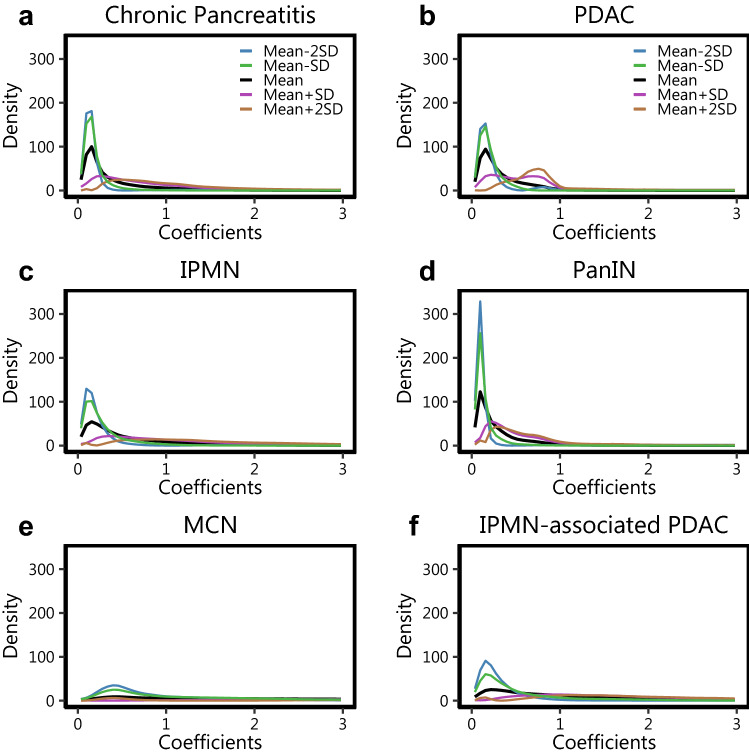
The first principal direction of variability for all images in each of the disease cohorts. In each case we present the path sampled with standard deviations around the Karcher Mean of each disease cohort along the first principal component direction after dimension reduction.

Additionally, we wanted to observe the variation in the distribution of PDFs across all 6 groups, and their influence on the performance of the classification model. To facilitate this, we plot the path sampled by the PDFs with − 2, − 1, 0, + 1, + 2 standard deviations around the Karcher mean PDF along the first principal component direction in Fig. 3. Though we observe that the sample paths of CP (Fig. 3a) and PanIN (Fig. 3d) overlap with that of PDAC (Fig. 3b), the distribution of these paths across their sample space is fairly distinct. This observation corroborates with the AUCs observed between CP and PDAC, and PanIN and PDAC. It is seen that the peaks for PDAC are at different locations along the path as opposed to the ones observed for CP and PanIN. In contrast, the sample paths for CP and PanIN have a strong overlap , explaining the relatively poor performance of the corresponding pairwise classifier. It is also observed that of all the pairwise classifiers, MCN is the most difficult to be discriminated from, when looked at from a disease group context. The result might be influenced by the comparatively low sample size of the MCN cohort.

To validate the high discriminatory potential between CP and PDAC, we applied the GaWRDenMap framework on an anonymized independent validation cohort. This consisted of one image from each patient, obtained for 7 patients from a CP cohort and 7 from a PDAC cohort. Classification metrics for the model were computed, and the AUC obtained was 0.715 ($95\%$ CI: 0.415-1) with sensitivity as 0.75 and specificity as 0.857. The wide range of the confidence interval and the lower AUC can be explained by the smaller sample size of the validation cohort. Nevertheless, we observe reasonably high AUC, sensitivity and specificity values using GaWRDenMap on this small validation cohort.

## Discussion

Though there are different methods to capture the spatial heterogeneity in the microenvironment, many only provide a single-number summary (e.g. Morisita-Horn index^20^) to explain the variation in cell-cell interactions across the field of view. Our GaWRDenMap framework aims to capture the heterogeneity in the field of observation in a spatially-informed manner by providing a function to do the same,namely the PDFs of the GWR coefficients, and leverages the quantification of spatial heterogeneity as a potential biomarker to differentiate between the different disease groups. Our method was applied to a cohort consisting of 6 different groups of pancreatic diseases, and demonstrated reasonable discriminative ability between the spatial arrangement of specific cell phenotypes of any two groups. For pairwise comparison to be deemed significant in our study, an AUC value of 0.75 was determined to be the cutoff. This was determined after analysing the results as a whole and on advice from the physician. Results show that our GaRWDenMap framework displays better classification performance as opposed to the simple summary statistics-based spatial methods such as the Morisita-Horn Index, which was considered as baseline for our study. We infer that our framework is able to make use of richer spatial information from the observed tissue to better differentiate between different diseases afflicting the same organ.

Of all the pairwise comparisons between the six groups, the ability to distinguish between CP and PDAC has particular relevance from both clinical and pathological perspectives. CP has been shown to have a greater chance of developing into PDAC, but it is usually challenging for the pathologists to discern between the two, based on arrangement and structure of the cells^25,26^. This can result in under-treatment of cancers and morbid surgical resection in patients with non-neoplastic pancreatitis. Diagnostic uncertainty can also lead to repeated biopsies with associated morbidity and delay in treatment. On our and related work, the spatial arrangement might have a role to play for the grade of disease, thus prompting investigation based on spatial modelling approaches for this classification problem. As illustrated in Figs. 2 and 3, there is an evident visual difference in the distribution of the density curves between the two groups of interest. The observed greater range of coefficient values through our analysis suggests a greater spatial heterogeneity between lymphocytes and epithelial cells across samples in PDAC as compared to CP. Previous studies have also shown the influence of the spatial arrangement of cells in IPMN in the progression to PDAC, with the interactions of epithelial cells with lymphocytes identified as an important correlate of dysplasia grade in IPMNs^14^. The results from GaWRDenMap point to a difference in the spatial arrangement of epithelial and immune cells between IPMN and PDAC. The higher discriminatory power of the framework also points towards greater attention to be paid to capture the difference in cellular spatial arrangement across the sample, as these patterns can provide more spatially-aware information. Our results thus show that the GaWRDenMap framework can provide an addition layer that is spatially informed, which is missed by visual observation of the mIF-stained pathological images. This also warrants further investigation of such spatial interactions varying across the observation sample, of similar cell phenotypes of interest present in the microenvironment.

Our analysis framework utilizes intensity measures from point patterns as the input to a GWR to construct the PDF signature. Our study considered two cell species for regression modelling, however, it can be easily extended to include more than two cell types. This potentially provides an opportunity to explore multi-way heterogeneity in cellular interactions. Of particular interest would be examining higher order cell interactions, for example, the influence of t-regulatory cells in the joint spatial arrangement of epithelial cells and cytotoxic lymphocytes. In its current form, our framework is used purely for classification purposes, but it is possible to further utilize the generated biomarker signals as a potential prognostic variable in diagnostic models, once value has been adequately demonstrated. The flexibility of the framework would not restrict it to the imaging domain; the set-up allows extension to other omics data with similar spatial characteristics, ranging from the output from a simple H&E slide, to higher dimensional spatial transcriptomics data. As a proposed example, it would be possible to map out gene expression patterns across sub-regions of the tissue under observation, and infer the variation in target gene expression based on the activation of other genes in the neighbourhood. From a broader perspective, the framework can provide an additional and/or alternative avenue to understand the biological differences in spatial interactions, between clinically distinct pathologies, which can be extended for use in a diagnostic setting. This can be useful in situations where standard pathological analysis leads to ambiguous conclusions that are frequently encountered when distinguishing pathologies with overlapping differential diagnoses.

Working with mIF images provides the specificity required to identify multiple cell phenotypes, but the processes involved are time-consuming and not easily available. The extension and optimization of this framework on the more widely available H&E-stained images is the next step in increasing the generalizability of the model for pathology image analysis applications. The variability in sample size between the groups is a limitation of this study, with patient sample sizes ranging from 16 for the MCN cohort to 71 for the PDAC cohort.Further analysis on a more balanced and comprehensive data set would be warranted to explore and validate some of the observations made in the results from this study cohort. Currently, our workflow is optimized for pairwise classification problems, but with the availability of a more balanced cohort it is possible to extend our framework to a multi-class classification setting.

To summarize, we developed a PDF-based classification model to differentiate between six pancreatic disease cohorts in this proof-of-concept study. This took into consideration the heterogeneity in interaction between two distinct cell phenotypes present in the microenvironment. Our method goes a step beyond previous abundance-based methods that are commonly used in the field. The density functions and in turn the classification model was able to strongly differentiate between CP and PDAC, and IPMN and PDAC, both of which are of clinical importance. Further investigation into the clinical implications of the results on a larger scale would be warranted. It is also important to acknowledge that a thorough exploration and validation is required before our framework can be deployed in a clinical setting. Optimized sample size estimation, error quantification in a cost-sensitive manner, and model uncertainty quantification need to be assessed to build trust in the model and for clinical deployment.

## Methods

Our first step involves generating the intensity functions from the point patterns of our cell phenotypes of interest, namely, epithelial and immune cells. Firstly, for each image/subject, we use the spatial locations of each cell phenotype to understand their individual distribution in the image by generating point intensity maps. Secondly, we use the generated intensity maps of epithelial and immune cells across all the images/subjects as predictor and response, respectively, via a GWR model. Thirdly, we construct a PDF using the GWR model coefficients, which serves as a signature for the epithelial-immune cell interaction. Finally, we use the PDFs across all the images/subjects to build classification models to discern between disease groups. Further details about each of these steps are provided next.

### Cell phenotype intensity estimation

For representation purposes, each cell on the image is represented by a point on a 2-dimensional grid, with the location determined from the staining framework mentioned in the “Data” section. Thus, the spatial locations and distribution of the cell phenotypes on a pathology slide is visualized through a point pattern representation on a grid, as is shown on Fig. 1. We consider a smoother representation of the distribution of the cell phenotype in the field of view. That is, we construct an intensity function separately for each cell type, using two-dimensional isotropic kernel intensity estimation by considering the cell locations as point patterns on a spatial grid. We select the grid dimensions to ensure that they are proportional to the image dimensions in pixels. The intensity estimate at a point *u* on the grid is given by, ${\widehat{\lambda }}(u) = e(u) \sum _i k(x_{i}-u)w_{i}$, where $x_i$ is the *i*th data point of the point pattern *x*, *k* is a Gaussian smoothing kernel, *e*(*u*) is an edge correction factor, and $w_i$ are the weights. The computed intensity is then corrected for edge effect bias by dividing it with the convolution of the Gaussian kernel with the window of observation^27^. This correction term is represented as $\frac{1}{e(u)} = \int _{W} k(v-u)\mathrm {d} v$, where *W* is the observation window^27^. Using the above approach, we compute the kernel intensity estimates for each of the two cell types. Computations were performed using the *spatstat*^28^ package in R software^29^. The corresponding epithelial and immune cell intensity values on the spatial grid are used as predictor and response in a GWR model. An example of the generated intensity surfaces from the respective phenotype point patterns is shown in Supplementary Fig. S1.

### GWR model

**Figure 4:**
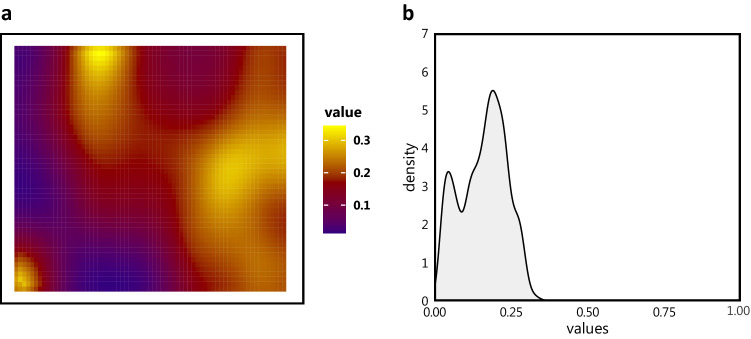
The map of an example GWR model coefficient as a surface (**a**) and the corresponding probability density function (**b**) constructed using these model coefficients.

GWR has been used as an exploratory tool to assess local changes in relationships between dependent and independent variables, especially for spatial point data^22^. It is an extension of the ordinary least squares regression, that incorporates spatial variability to the relationship between dependent and independent variables^22^. It can be represented as $y_{i} = \beta _{i0} + \beta _{i1}x_{i1} + \ldots + \beta _{ip}x_{ip} + \varepsilon _{i}$, where $\beta$ is the local coefficient of regression at a given location *i*, *y* is the response, *x* is the independent predictor, and $\varepsilon$ is the error. However, prior to the GWR model estimation, we need to consider the following preliminary steps: (a) creation of the polygonal spatial grid on which the model is to be estimated, and (b) estimation of the kernel bandwidth. To address (a), we consider the polygonal spatial grid based on the overlapping epithelial-immune region. For this spatial grid, we consider only the region where the epithelial kernel intensity is above a predetermined threshold, to avoid any ill-conditioned behavior. The bandwidth estimation can either be constant (fixed distance) or adaptive (fixed number of neighborhood samples). Since our data is irregularly sampled, to address (b), we utilize an adaptive kernel bandwidth which is chosen such that it minimizes the second order Akaike Information Criterion (AICc). AICc takes sample size into consideration, thus ensuring a better fit for the local regression model at any grid point^30^. We now fit our spatially-varying regression model on every point on the grid constructed on the overlapping region. The epithelial and immune intensity values are selected as the independent and dependent variables, respectively. The GWR coefficient estimates are obtained at each grid point in the window. An example for this is shown in Fig. 4. The GWR coefficient estimates are computed using the *GWModel* package^31,32^ in R software^29^.

### PDF-based classification model

The GWR coefficients allow us to observe spatially-varying changes in the interaction between the point patterns of epithelial and immune cells. After computing the GWR coefficients in the previous step, we assess the distribution of the coefficient values across the spatial grid. For this purpose, for each image/patient, we construct a PDF (via a one-dimensional kernel density estimation) using the estimated GWR coefficients. These PDFs can be interpreted as the epithelial-immune cell interaction ‘signature’. This PDF representation captures the distribution and frequency of occurrence of each value, and hence comprehensively summarizes the distribution of the GWR coefficients compared to extracting summary features using the model coefficients.

Given a sample of PDFs corresponding to a sample of subjects, we utilize the Riemannian-geometric framework which allows us to work with the PDFs as data objects^33–35^. This framework leverages the geometry of the space of PDFs to map densities, which are functional data objects, to a vector representation. The Riemannian-geometric framework, via the square-root transformations of the PDFs, provides important quantities of interest such as a distance metric between two PDFs, the average of a sample of PDFs, and a principal component analysis on a tangent space of the space of PDFs^36^. Further mathematical details are provided in the Supplementary Material. The principal component (PC) scores, obtained via a principal component analysis on PDFs, serve as Euclidean representations of the PDFs. We compute the PC scores for every disease group and use them as predictors in our downstream classification model. The PC scores computed from a sample of PDFs is a matrix, where every row corresponds to one sample and each column corresponds to the individual PC. The total number of PCs to include is determined as the number of PCs needed to represent 99.99% of total variation in the sample of PDFs.

We build a classification model to discern between two groups of interest using these PC scores as predictors. For this paper, we considered a probit regression model, a type of generalized linear model used to model binary outcome variables^37^. We utilize this regression model, as our response, *Y*, is binary with two possible outcomes. If *X* is assumed to be the predictor (which are the PC scores in this case) and *Y* is the binary response indicating membership to class 1 of the two classes in the problem, the model can be written as $Pr(Y=1 | X)=\phi (X^T \beta )$, where $\phi$ is the cumulative density function of the standard normal distribution, and $\beta$ is a parameter estimated by standard estimation methods such as maximum likelihood. We train this probit model using the PC scores from our training data as predictors. For our test data, we use the Riemannian-geometric framework to map the PDFs to a vector of PC scores. We then use the trained model to classify the test samples. Leave-one-out-cross validation was used to validate the performance of our model for every binary classification pair. The area under the receiver-operator characteristics curve (AUC) and the 95% confidence interval(CI), sensitivity and specificity of each model was also reported.

### Approval, accordance and informed consent

This study involving human subjects was approved by University of Michigan Institutional Review Board and conducted in accordance to the Declaration of Helsinki. All patients signed an informed consent form prior to enrollment.

## Supplementary Information

Supplementary Information.

## Data Availability

The datasets used in this study are available from the University of Michigan School of Medicine, but are not publicly accessible due to restrictions on licensing for use by the institution. The datasets may be available from the corresponding author on reasonable request, with appropriate permissions from the University of Michigan School of Medicine.
