# Supplementary material for: GaWRDenMap: a quantitative framework to study the local variation in cell–cell interactions in pancreatic disease subtypes
Source: Sci Rep. 2022 Mar 8;12:3708. doi: 10.1038/s41598-022-06602-z (PMC8904504; doi:10.1038/s41598-022-06602-z)
Supplement: Supplementary file 1 — Supplementary Information. [file 41598_2022_6602_MOESM1_ESM.pdf]

Supplementary material for "GaWRDenMap : A Quantitative Framework to study the local variation in tumor-immune interactions in Pancreatic disease subtypes"

Santhoshi N. Krishnan[1,2,+,*], Shariq Mohammed[3,4,+], Timothy L. Frankel[5], and Arvind Rao[1,2,3,6,7,*]

[1]Department of Electrical and Computer Engineering, Rice University, Houston, TX, USA
[2]Department of Computational Medicine and Bioinformatics, University of Michigan, Ann Arbor, MI, USA
[3]Department of Biostatistics, University of Michigan, Ann Arbor, MI, USA
[4]Department of Biostatistics, Boston University, Boston,MA, USA
[5]Department of Surgery, University of Michigan, Ann Arbor, MI, USA
[6]Department of Radiation Oncology, University of Michigan, Ann Arbor, MI, USA
[7]Department of Biomedical Engineering, University of Michigan, Ann Arbor, MI, USA
[*]ukarvind@umich.edu
[+]these authors contributed equally to this work

ABSTRACT

We describe the mathematical details for the geometric framework to compute distance, mean and principal component analysis using a sample of probability density functions as data objects. We also give a brief description of the Morisita-Horn index, and the parameters used for it's implementation on our data. Additionally, we also include supplementary images referenced in the manuscript.

Space of probability density functions and their transformations

Given a sample of PDFs corresponding to a sample of subjects, we utilize the Riemannian-geometric framework which allows us to work with the PDFs as data objects. This has been used in several applications in the context of analyzing tumor heterogeneity from radiology images in gliomas[1–3]. Here we describe the relevant mathematical details.

PDFs belong to a non-linear functional space and we exploit the differential geometry of this space. For this discussion and brevity, we restrict ourselves to the case of univariate densities on $[0,1]$ and normalize the PDFs so that they belong to the Banach manifold $\mathscr{F} = \{f : [0,1] \to \mathbb{R}_{\geq 0} | \int_0^1 f(x)dx = 1\}$. For any point f a tangent space, which is a vector space of all possible perturbations of f, can be defined as $T_f(\mathscr{F}) = \{\delta f : [0,1] \to \mathbb{R} | \int_0^1 \delta f(x)dx = 0\}$. A Reimannian metric on $\mathscr{F}$ such as the non-parametric Fisher-Rao metric (FR metric) can be used to compute geodesic distances between two PDFs and also the summary statistics of a sample of PDFs[4–6].Although FR metric has nice mathematical properties it changes from point to point on $\mathscr{F}$.

We transform the PDFs such that the complex non-linear space changes to a computationally much simpler space. We consider the square-root transformation (SRT), $h = +\sqrt{f}$[7], since it measures the distance between any two points in $\mathscr{F}$ as a standard $\mathbb{L}^2$ Riemannian metric[8]. We omit the '+' sign hereafter for notational convenience. The space of the SRTs corresponding to $\mathscr{F}$ is given by $\mathscr{H} = \{h : [0,1] \to \mathbb{R}_{\geq 0} | \int_0^1 h^2(x)dx = 1\}$, which is the positive orthant of the unit Hilbert space[9]. The tangent space at any point $h \in \mathscr{H}$ is defined as $T_h(\mathscr{H}) = \{\delta h : [0,1] \to \mathbb{R} | \int_0^1 h(x)\delta h(x)dx = 0\}$. The geodesic distance between two densities $f_1, f_2 \in \mathscr{F}$, represented by their SRTs $h_1, h_2 \in \mathscr{H}$, is defined as the shortest arc connecting them on $\mathscr{H}$, that is, $d(h_1,h_2) = \theta := cos^{-1}\left(\int_0^1 h_1(x)h_2(x)dx\right)$. This is also the standard $\mathbb{L}^2$ distance between $h_1, h_2 \in \mathscr{H}$.

Let f_i denote the PDF for subject i for all $i = 1,\ldots,n$ and $h_1,\ldots,h_n$ be their corresponding SRTs. A generalized version of the mean on a metric space that can be used to compute the average density is called the Karcher mean[10]. Specifically, as the unique inverse transformation of the SRT is given by $f = h^2$[8], the sample average of PDFs $f_1,\ldots,f_n$ can be computed as $\bar{f} = \bar{h}^2$, where $\bar{h}$ is the sample average on the space of SRTs. The sample Karcher mean $\bar{h}$ on $\mathscr{H}$ is the minimizer of the Karcher variance $\rho(\bar{h}) = \sum_{i=1}^n d(\bar{h}, h_i)^2_{\mathbb{L}^2}$, that is, $\bar{h} = \text{argmin}_{h \in \mathscr{H}} \rho(\bar{h})$. Algorithm 1 presents a gradient-based approach to compute

the Karcher mean on Ψ[11,12]. The computations require important tools from differential geometry called the exponential and inverse-exponential maps. For $h \in \mathscr{H}$ and $\delta h \in T_h(\mathscr{H})$, the exponential map at h, $\exp : T_h(\mathscr{H}) \to \mathscr{H}$ is defined as $\exp_h(\delta h) = cos(||\delta h||)h + sin(||\delta h||)\delta h/||\delta h||$, where $||\delta h||^2 = \int_0^1 \left(\delta h(x)\right)^2 dx$. For any $h_1, h_2 \in \mathscr{H}$, the inverse-exponential map is denoted by $\exp_{h_1}^{-1} : \mathscr{H} \to T_{h_1}(\mathscr{H})$ and is defined as $\exp_{h_1}^{-1}(h_2) = \theta[h_2 - cos(\theta)h_1]/sin(\theta)$.

Algorithm 1 Sample mean on the space of SRTs

1: $\bar{h}_0$ (initial estimate for the Karcher mean) $\leftarrow$ any one of the densities in the sample OR the extrinsic average. Set $j \leftarrow 0$ and $\varepsilon_1, \varepsilon_2 > 0$ be small.
2: For $i = 1, \ldots, n$ compute $u_i = \exp_{\bar{h}_j}^{-1}(h_i)$.
3: Compute the average direction in the tangent space $\bar{u} = \frac{1}{n}\sum_{i=1}^{n} u_i$.
4: **if** $||\bar{u}||_{L^2} < \varepsilon_1$ **then**
5:     **return** $\bar{h}_j$ as the Karcher mean.
6: **else**
7:     $\bar{h}_{j+1} = \exp_{\bar{h}_j}(\varepsilon_2 \bar{u})$.
8:     Set $j \leftarrow j+1$.
9:     Return to step 2.
10: **end if**

Note that the tangent space, $T_{\bar{h}}(\mathscr{H})$, is a vector (Euclidean) space, hence PCA can be implemented as in standard problems. The implementation of PCA on the tangent space at the mean of SRTs $h_1, \ldots, h_n$ (corresponding to PDFs $f_1, \ldots, f_n$) is outlined in Algorithm 2. Here the orthogonal matrix U contains the principal components (PC), and the diagonal matrix Σ contains the PC variances. Note that the first r columns of U (denoted as $\tilde{U} \in \mathbb{R}^{m \times r}$) span the r-dimensional principal subspace. The choice of r could be made based on the cumulative amount of variance explained by the first few principal components. We can express the data using coordinates in this subspace via principal coefficients computed as $X = V\tilde{U}$, where $V^\top = [v_1\ v_2\ \ldots\ v_n] \in \mathbb{R}^{m \times n}$. These principal coefficients X (PC scores) act as Euclidean coordinates corresponding to densities and can be used as predictors for downstream analysis.

Algorithm 2 PCA on $T_{\bar{h}}(\mathscr{H})$

1: Compute the Karcher mean of $h_1, \ldots, h_n$ as $\bar{h}$ using Algorithm 1.
2: **for** $i = 1, \ldots, n$ **do**
3:     Compute projections of h_i onto $T_{\bar{h}}(\mathscr{H})$, that is, $v_i = \exp_{\bar{h}}^{-1}(h_i)$.
4: **end for**
5: Evaluate sample covariance matrix $K = \frac{1}{n-1}\sum_{i=1}^{n} v_i v_i^\top \in \mathbb{R}^{m \times m}$.
6: Compute the SVD of $K = U\Sigma U^\top$.

The Morisita-Horn Dissimilarity Index

The Morisita-horn dissimilarity index is a popular measure of overlap originally proposed and used in ecological and environmental modelling, where it was used to study predator-prey relationships in the observed environment[13]. The output values of these abundance-based similarity indices range from 0 to 1, with 0 indicating no overlap between the two cell species of interest, and 1 indicating perfect overlap of cells of both species[14]. This computation of "overlap" is done over a window of observation or "tessellation" defined by the user. Mathematically, a simplified representation of the index calculated between two population vectors $(c_1, c_2)\mathbb{N}_{\geq 0}^m \times \mathbb{N}_{\geq 0}$ can be defined as $MH(p_1, p_2) = (2p_1p_2)/(p_1^2 + p_2^2)$, where p_1 and p_2 are the corresponding normalized populations of our two species 1 and 2 of interest[15].

This metric has been used and shown in prior studies to be a significant factor for breast cancer[16].
For our paper, we selected the quadrant window size for computing the index value to be a 250 x 250 microns square. This value was selected from the original paper by Maley et.al. that first introduced this concept . The species being compared were defined in the same way as they were during the the implementation of GaWRDenMap. All computations and analyses were implemented using R (R Core Team (2013)).

Supplementary Images

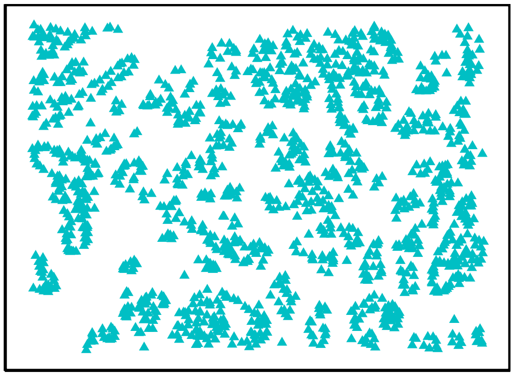

(a) The point pattern representation of Epithelial cells

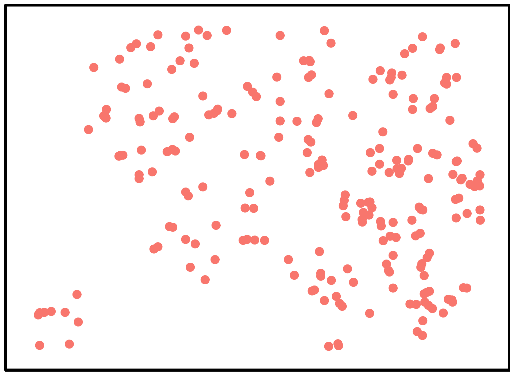

(b) The point pattern representation of Immune cells

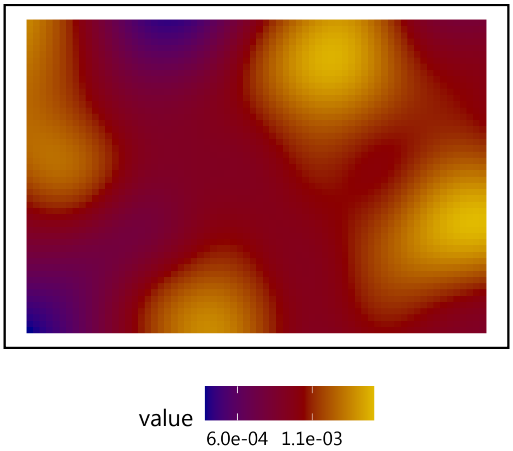

(c) The corresponding epithelial intensity grid

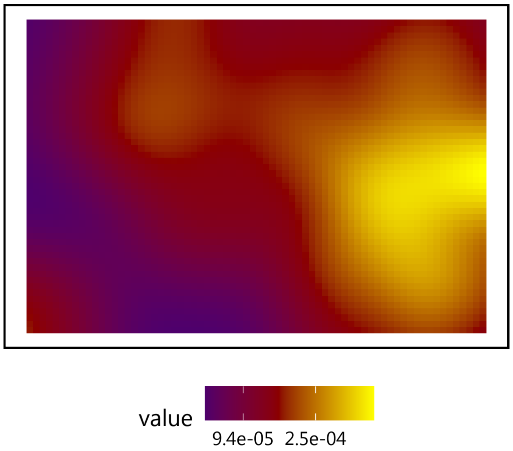

(d) The corresponding immune intensity grid

Figure S1. Representatives figures from one image in the cohort depicting the individual epithelial and immune cell point patterns (top), and the corresponding intensity surfaces (bottom).

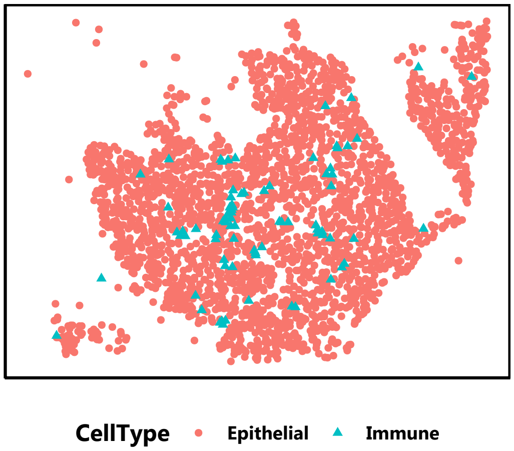

(a) The point pattern representation of tumor and immune cells

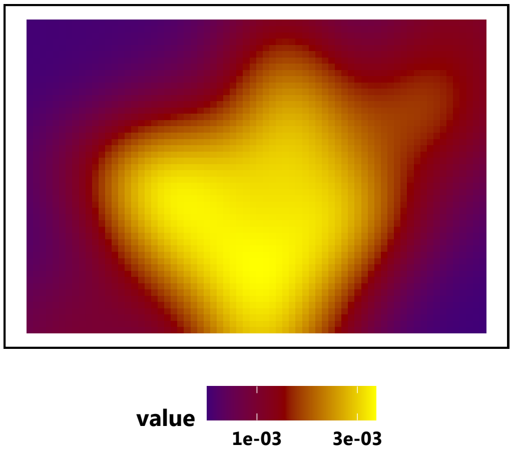

(b) The tumor intensity image before thresholding

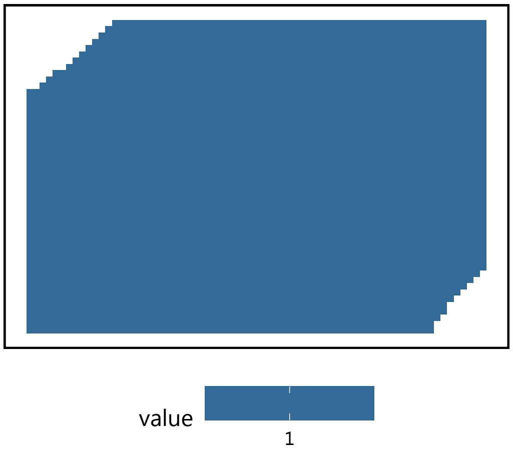

(c) The overlay mask grid for thresholding

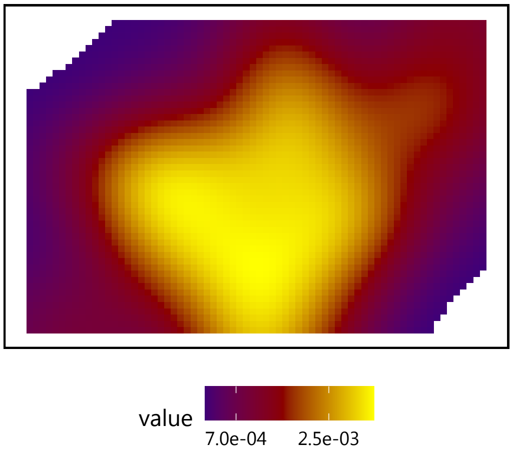

(d) Tumor intensity image after using the mask grid

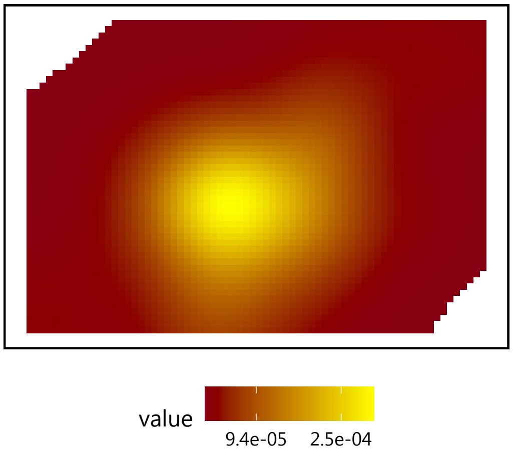

(e) Immune intensity image after using the mask grid

Figure S2. An example for when a threshold mask is used to remove regions where the intensity surface of the independent variable (in our case, the tumor/epithelial cell intensity shown in S2b) is very low. This is done for images where the point pattern is sparse and/or not enough representative points are present, to avoid extreme outlier GWR values. For our study, a value on 0.0001 was selected after observing the spread of the density values across all images in the cohort, to create a mask (S2c). This mask is applied on the original tumor intensity image, and only the regions falling within the mask are retained (S2d). The same mask is applied on immune intensity image as well (S2e). These obtained regions are then used to compute the coefficients.

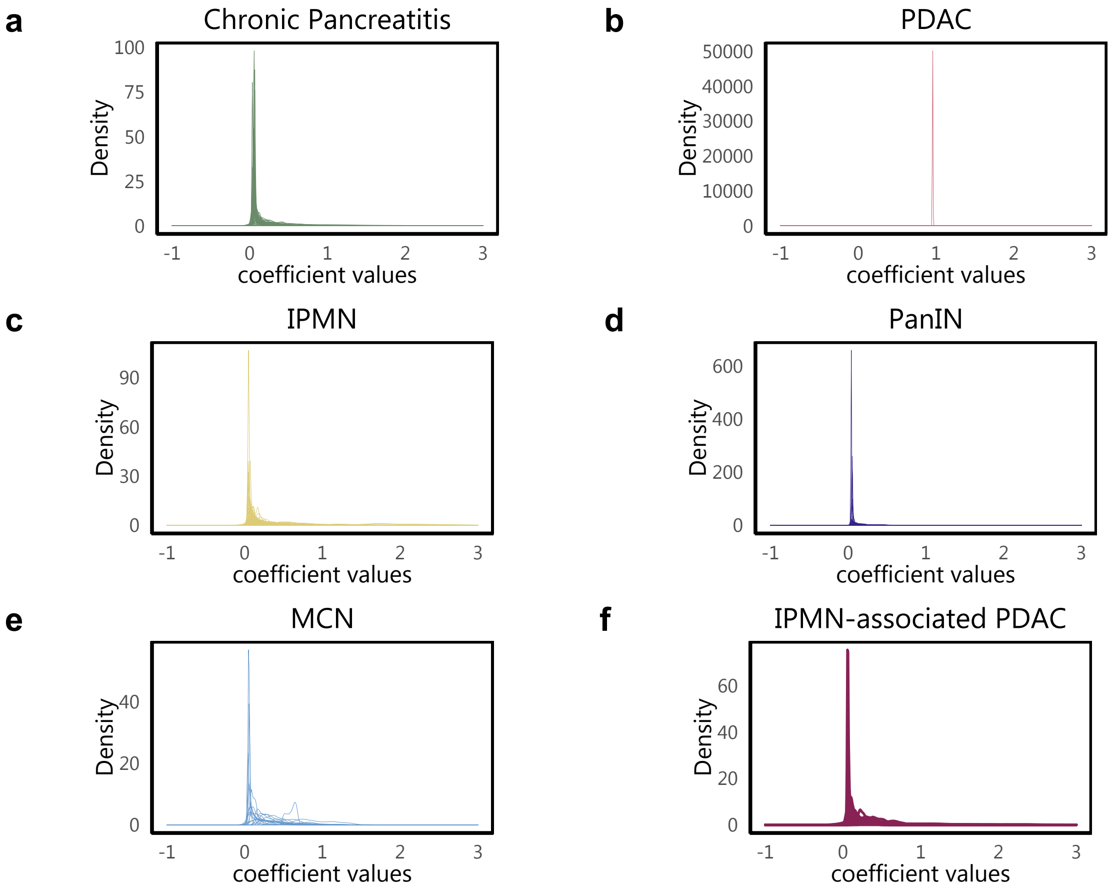

Figure S3. The complete, untruncated density estimates for all subjects across all six pancreatic disease sub types. Here, the x-axis corresponds the GWR coefficient values obtained at every point on our GWR computation grid.

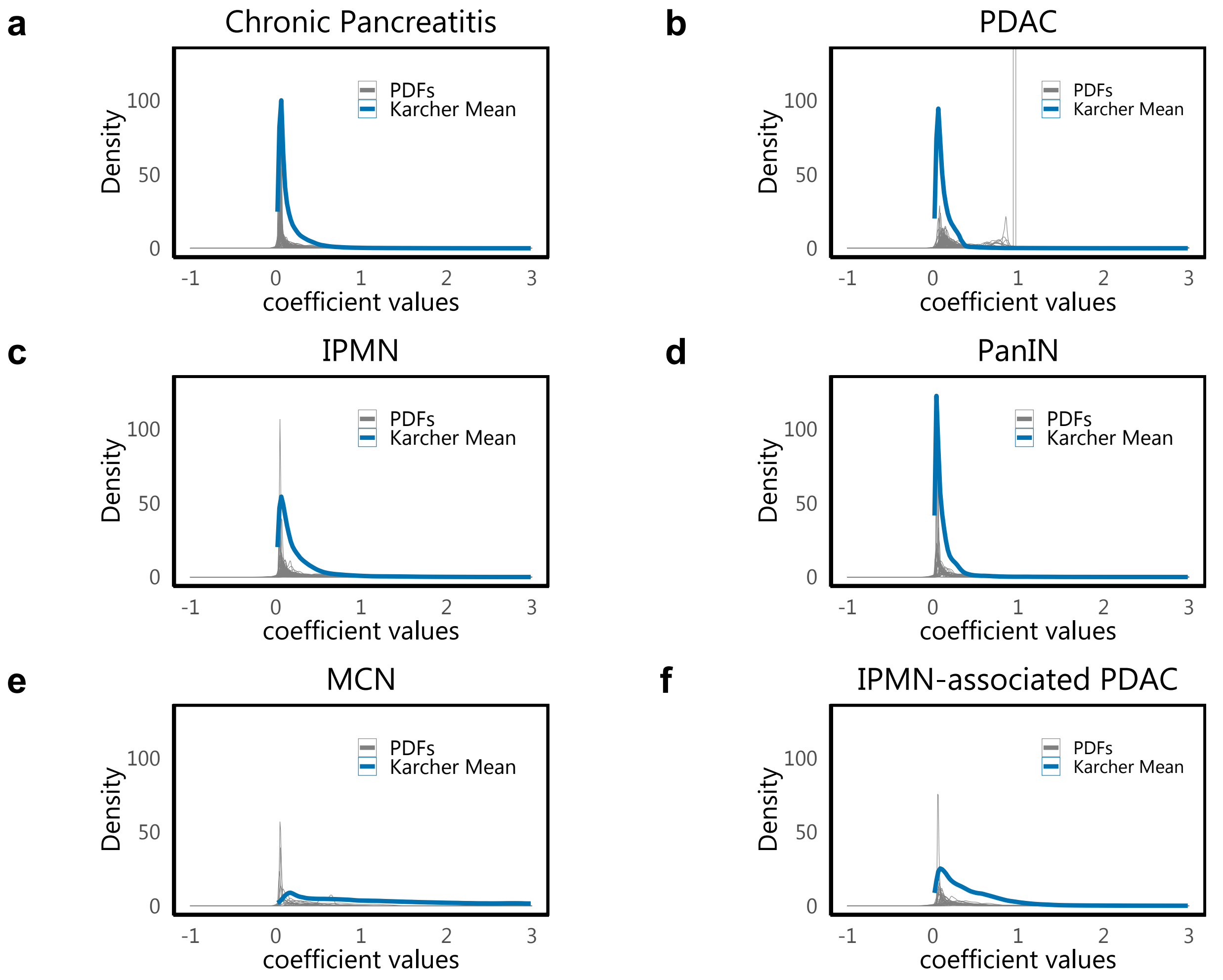

Figure S4. The Karcher means of all 6 diseases groups overlaid over the PDFs. Here, the PDFs are truncated to eliminate outliers in order to observe the Karcher Mean in perspective. The x-axis corresponds the GWR coefficient values obtained at every point on our GWR computation grid, and the y-axis corresponds to the density of coefficient values.

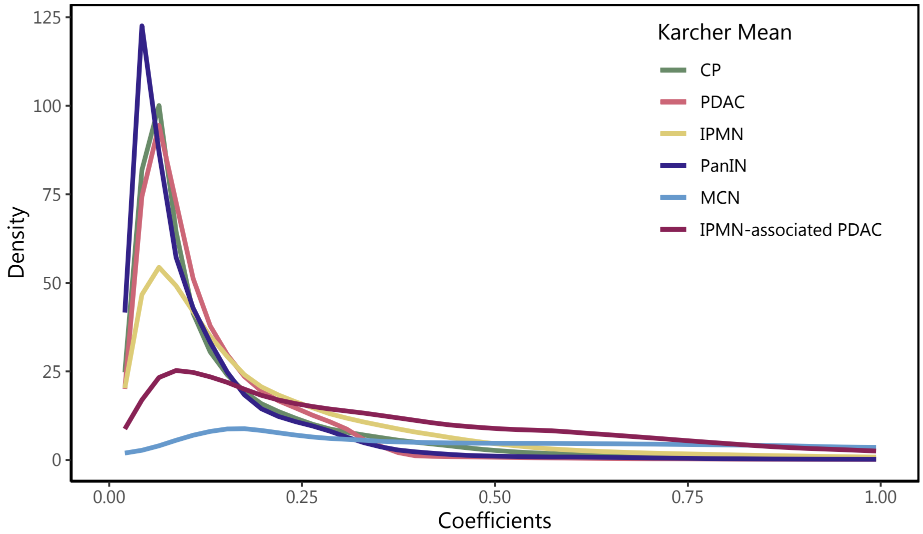

Figure S5. The Karcher means of all 6 diseases groups overlaid on each other. We can observe that there is a difference in the range and occurrence of specific GWR coefficients between each of the disease groups, with CP displaying the highest peak of all the diseases, and MCN with the lowest.

References

1. Matuk, J., Mohammed, S., Kurtek, S. & Bharath, K. Biomedical applications of geometric functional data analysis. *Handb. Var. Methods for Nonlinear Geom. Data* 675–701, DOI: 10.1007/978-3-030-31351-7_24 (2020).
2. Mohammed, S. *et al.* Density-based classification in diabetic retinopathy through thickness of retinal layers from optical coherence tomography. *Sci. Reports* **10**, DOI: 10.1038/s41598-020-72813-x (2020).
3. Mohammed, S., Bharath, K., Kurtek, S., Rao, A. & Baladandayuthapani, V. RADIOHEAD: Radiogenomic Analysis Incorporating Tumor Heterogeneity in Imaging Through Densities. *arXiv preprint arXiv:2104.00510* (2021).
4. Rao, C. R. Information and the accuracy attainable in the estimation of statistical parameters. In *Breakthroughs in Statistics*, 235–247 (Springer, 1992).
5. Kass, R. E. & Vos, P. W. *Geometrical foundations of asymptotic inference*, vol. 908 (John Wiley & Sons, 2011).
6. Srivastava, A., Jermyn, I. & Joshi, S. Riemannian analysis of probability density functions with applications in vision. In *2007 IEEE Conference on Computer Vision and Pattern Recognition*, 1–8 (IEEE, 2007).
7. Bhattacharyya, A. On a measure of divergence between two statistical populations defined by their probability distributions. *Bull. Calcutta Math. Soc.* **35**, 99–109 (1943).
8. Kurtek, S. & Bharath, K. Bayesian sensitivity analysis with the fisher–rao metric. *Biometrika* **102**, 601–616 (2015).
9. Lang, S. *Fundamentals of differential geometry*, vol. 191 (Springer Science & Business Media, 2012).
10. Karcher, H. Riemannian center of mass and mollifier smoothing. *Commun. on pure applied mathematics* **30**, 509–541 (1977).
11. Dryden, I. & Mardia, K. *Statistical analysis of shape* (Wiley, 1998).
12. Kurtek, S. *et al.* A geometric approach to pairwise bayesian alignment of functional data using importance sampling. *Electron. J. Stat.* **11**, 502–531 (2017).
13. Wolda, H. Similarity indices, sample size and diversity. *Oecologia* **50**, 296–302, DOI: 10.1007/bf00344966 (1981).
14. Magurran, A. E. Biological diversity. *Curr. Biol.* **15**, R116–R118 (2005).

15. Rempala, G. A. & Seweryn, M. Methods for diversity and overlap analysis in t-cell receptor populations. *J. mathematical biology* **67**, 1339–1368 (2013).

16. Maley, C. C., Koelble, K., Natrajan, R., Aktipis, A. & Yuan, Y. An ecological measure of immune-cancer colocalization as a prognostic factor for breast cancer. *Breast Cancer Res.* **17**, DOI: 10.1186/s13058-015-0638-4 (2015).
